# Effects of Incretin-Related Diabetes Drugs on Bone Formation and Bone Resorption

**DOI:** 10.3390/ijms22126578

**Published:** 2021-06-19

**Authors:** Hideki Kitaura, Saika Ogawa, Fumitoshi Ohori, Takahiro Noguchi, Aseel Marahleh, Yasuhiko Nara, Adya Pramusita, Ria Kinjo, Jinghan Ma, Kayoko Kanou, Itaru Mizoguchi

**Affiliations:** Division of Orthodontics and Dentofacial Orthopedics, Graduate School of Dentistry, Tohoku University, 4-1, Seiryo-machi, Aoba-ku, Miyagi, Sendai 980-8575, Japan; saika.ogawa.d2@tohoku.ac.jp (S.O.); fumitoshi.ohori.t3@dc.tohoku.ac.jp (F.O.); takahiro.noguchi.r4@dc.tohoku.ac.jp (T.N.); marahleh.aseel.mahmoud.t6@dc.tohoku.ac.jp (A.M.); yasuhiko.nara.q6@dc.tohoku.ac.jp (Y.N.); adya.pramusita.q6@dc.tohoku.ac.jp (A.P.); ria.kinjou.p5@dc.tohoku.ac.jp (R.K.); ma.jinghan.s1@dc.tohoku.ac.jp (J.M.); kanou.kayoko.s7@dc.tohoku.ac.jp (K.K.); mizo@tohoku.ac.jp (I.M.)

**Keywords:** osteoclast, osteoblast, GLP-1, DPP-4, diabetes, GLP-1 receptor agonist, DPP-4 inhibitor

## Abstract

Patients with type 2 diabetes have an increased risk of fracture compared to the general population. Glucose absorption is accelerated by incretin hormones, which induce insulin secretion from the pancreas. The level of the incretin hormone, glucagon-like peptide-1 (GLP-1), shows an immediate postprandial increase, and the circulating level of intact GLP-1 is reduced rapidly by dipeptidyl peptidase-4 (DPP-4)-mediated inactivation. Therefore, GLP-1 receptor agonists and DPP-4 inhibitors are effective in the treatment of type 2 diabetes. However, these incretin-related diabetic agents have been reported to affect bone metabolism, including bone formation and resorption. These agents enhance the expression of bone markers, and have been applied to improve bone quality and bone density. In addition, they have been reported to suppress chronic inflammation and reduce the levels of inflammatory cytokine expression. Previously, we reported that these incretin-related agents inhibited both the expression of inflammatory cytokines and inflammation-induced bone resorption. This review presents an overview of current knowledge regarding the effects of incretin-related diabetes drugs on osteoblast differentiation and bone formation as well as osteoclast differentiation and bone resorption. The mechanisms by which incretin-related diabetes drugs regulate bone formation and bone resorption are also discussed.

## 1. Introduction

Diabetes is associated with a number of musculoskeletal complications, including increased incidences of bone fracture and osteoarthritis, which lead to joint pain and loss of function [1,2,3,4]. Although type 2 diabetes patients have high bone mineral density (BMD), they have a higher fracture risk than the general population [5,6,7,8,9]. The reasons for this increased fracture risk are multifactorial, and include increased fall risk, changes associated with chronic hyperglycemia, and diabetes-related microvascular complications [10]. There is a linear relation between serum hemoglobin A1c (HbA1c) level and risk of hip fracture in patients with type 2 diabetes, and the risk of hip fracture was shown to be higher in patients with HbA1c level >9% than 6–7% [6]. Several studies also showed that patients with inadequate glycemic control have a high risk of fracture [11,12,13]. However, a randomized clinical trial indicated little difference in the effect on risk of fracture between intensive treatment, with a median HbA1c of 6.4%, and normal treatment, with a median of HbA1c 7.5%, in patients with type 2 diabetes [14]. However, it has also been reported that intensive control of hyperglycemia is related to elevated risk of fracture [15]. Many studies have suggested that some pathophysiological alterations in chronic hyperglycemia may be related to defects in bone formation [16,17]. For example, advanced glycation end products (AGEs) have been shown to inhibit osteoblast differentiation [18], osteoblast function [19], and attachment to collagen matrix [20], as well as increasing sclerostin expression in osteocytes [21,22,23]. Furthermore, chronic hyperglycemia may induce deposition of AGEs in bone collagen, resulting in a reduction in bone quality [24,25] and increased fracture risk [26,27]. Spontaneously diabetic rats may have altered vitamin D metabolism and impaired active intestinal calcium absorption, resulting in impaired bone mineralization [28]. Furthermore, glucose was shown to inhibit calcium uptake in osteoblasts in culture, resulting in inhibition of bone nodule formation [29]. Type 2 diabetes was shown to be associated with reduced levels of bone formation markers [30] and histomorphometric analysis demonstrated a decreased bone formation rate in diabetic patients [31], indicating reduced osteoblast function and skeletal dynamics in these patients. Osteoblasts are differentiated from mesenchymal stem cells and are important in bone formation. Bone metabolism is determined by the balance of bone formation and bone resorption [32]. Several studies have shown that hyperglycemia affects osteoblast function and differentiation both directly and indirectly [33,34,35,36,37,38,39]. In addition, hyperglycemia is known to enhance the adipogenic pathway of bone marrow-derived progenitor cells and suppress the induction of differentiation into osteoblast progenitor cells via inducing the expression of peroxisome proliferator-activated receptor-γ (PPAR-γ), resulting in suppression of bone formation [35,40,41]. Increases in lipid levels produced by hypoxic adipose tissue indirectly reduce osteoblast function via the release of cytokines, such as TNF-α and IL-6 [35,42,43]. A previous systematic review showed that diabetic condition negatively affects bone formation in animal models [44]. However, the mechanism by which diabetes affects the skeletal system is still only partially understood.

In the bone remodeling process, bone resorption and formation are mediated by osteoclasts and osteoblasts, respectively [45]. Osteoclasts, which are differentiated from hematopoietic stem cells, have bone-resorbing activities [46]. Two factors, i.e., receptor activator of nuclear factor-κB ligand (RANKL) and macrophage colony-stimulating factor (M-CSF), are important for osteoclast differentiation [47]. RANKL has been shown to induce osteoclast differentiation by binding to its receptor, receptor activator of nuclear factor-κB (RANK), on the surface of osteoclast precursor cells [48], while tumor necrosis factor-α (TNF-α) was reported to directly induce osteoclast formation independent of the RANKL/RANK pathway [49]. TNF-α may be related to the RANKL pathway in osteocytes [50]. Increasing adipose tissue by hyperglycemia induced expression of TNF-α and IL-6 [35,42,43]. These cytokines were shown to induce the formation and activation of osteoclasts. Hyperglycemia also enhances RANKL expression [42,51,52,53]. Hyperglycemia was shown to directly alter the expression of osteoblast-related genes, such as osteocalcin, and to affect osteoblast differentiation in vitro [54,55]. AGEs increase the expression of sclerostin in osteocytes in vitro, which inhibits bone formation [16,56]. Taken together, these results indicated that hyperglycemia inhibits bone formation and induces bone resorption.

Intestinal hormones accelerate the absorption of glucose by inducing the secretion of insulin from the pancreas. The observation that enteral nutrition stimulated a stronger insulin secretion response compared to intravenous sugar administration suggested the existence of incretins as insulin-inducing factors stimulated by alimentary glucose [57]. The first incretin, glucose-dependent insulinotropic polypeptide (GIP), was identified in porcine intestinal extract and was shown to have little effect on gastric acid secretion but to markedly upregulate insulin secretion in humans [58]. Glucagon-like peptide-1 (GLP-1) is an incretin hormone first identified by cloning the cDNA of the gene encoding proglucagon. Most GLP-1 is produced by intestinal L cells present in the distal ileum and colon, and the plasma GLP-1 level shows an immediate postprandial increase. As the circulating levels of both intact GLP-1 and GIP decline rapidly due to inactivation by the membrane-anchored enzyme peptidase, dipeptidyl peptidase-4 (DPP-4; also known as the lymphocyte cell-surface marker CD26), GLP-1 receptor (GLP-1R) agonists that mimic GLP-1 but are resistant to degradation by DPP-4 have been widely used in the treatment of type 2 diabetes [59,60,61]. DPP-4 inhibitors, which are used as diabetes drugs, block the enzymatic activity of DPP-4, therefore inhibiting degradation of the incretin hormones responsible for stimulating insulin secretion as a means of controlling blood glucose level [62]. A position statement published more than a decade ago by the American Diabetes Association (ADA) and the European Association for the Study of Diabetes (EASD) approved the use of DPP-4 inhibitors and GLP-1R agonists in treatment of type 2 diabetes and as second-line agents for treatment of poor glycemic control [63]. We showed previously that the DPP-4 inhibitor, linagliptin, and the GLP-1R agonist, exendin-4, attenuated osteoclast formation and bone resorption in vivo in a mouse model of lipopolysaccharide (LPS)-induced inflammation [64,65], and both of these agents were shown to inhibit mechanical stress-induced osteoclast formation and bone resorption in a mouse model of orthodontic tooth movement [66,67].

This review presents an outline of current knowledge regarding the effects of incretin-related diabetes drugs on osteoblast and osteoclast formation. We further discuss the possible mechanisms by which incretin-related diabetes drugs may regulate bone formation and resorption.

## 2. Effects of Incretin on Bone Formation

Incretins have long been known to be involved in insulin production, and the underlying mechanisms of these relations have been elucidated in some detail. Gutniak et al. [68,69] and Nathan et al. [68,69] evaluated the effects of GLP-1 in both type 2 diabetes patients and healthy nondiabetic controls, and a number of GLP-1R agonists (i.e., the short-acting exendin-4-based agonists exenatide and lixisenatide and the long-acting agonists albiglutide, dulaglutide, exenatide-LAR, and liraglutide) and DPP-4 inhibitors (i.e., sitagliptin, vildagliptin, saxagliptin, linagliptin, and alogliptin) have been used for the treatment of poor glycemic control in patients with type 2 diabetes [63,70]. The results of these studies demonstrated that GLP-1R agonists and DPP-4 inhibitors significantly upregulate the insulin response and can improve hyperglycemia in patients with type 2 diabetes.

GLP-1 and GIP are the two primary incretins released from the intestine induced by nutrient intake and exert their action via signaling mediated by incretin receptors [71,72]. GLP-1 is secreted by enteroendocrine cells of the intestinal mucosa, but GLP-1Rs are widely expressed on a number of cell types, including islet β-cells, and induce metabolic effects in various organs [73,74]. However, GLP-1 is degraded rapidly by DPP-4, which is a serine protease present in plasma, in many tissues [74,75,76]. GLP-1Rs are also expressed in bone-related cells where they induce a number of effects [73,77,78].

GIPRs are expressed on the osteosarcoma cell lines ROS 17/2.8, Saos-2, MG63, and TE-85 [78,79], mouse MC3T3-E1 [77], and mouse osteoblasts and human osteoblasts reflecting various stages of osteoblast differentiation [80,81]. Stimulation by GIP has been shown to reduce apoptosis of osteoblasts in vitro [80,81]. Stimulation of the GIP receptor (GIPR) on osteoblasts induces increases in intracellular cAMP levels, cell viability, alkaline phosphatase activity, and type 1 collagen expression [78,79]. However, daily injections of GIP did not improve cortical BMD, bone thickness, or bone strength in streptozotocin-induced diabetic mice, but experiments using the nanoindentation test showed that GIP can improve the mechanical properties of bone [82]. Administration of GIP and GLP-1 did not decrease the serum level of the bone resorption marker carboxy-terminal collagen crosslinks (CTX), also known as C-terminal telopeptide, although injection of GLP-2 decreased the serum CTX level [83]. Xie et al. examined the effects of GIP and its receptors on bone metabolism by dual-energy X-ray absorption and micro-computed tomography (μCT), and the results showed that GIPR knockout (KO) mice generated by targeted disruption of exons 4 and 5 of the GIPR gene encoding a portion of the extracellular domain have reduced total and femoral BMD as well as defects in trabecular structure and microarchitecture compared to wild-type controls [84]. On the other hand, Gaudin-Audrain reported that μCT analysis revealed increased number and volume of trabeculae in 16-week-old GIPR KO mice generated by deletion of the first six exons of the *GIPR* gene encoding the whole of the extracellular domain and a portion of the first transmembrane helix [85]. Mieczkowska et al. reported reductions of both bone strength and quality in a different strain of GIPR KO mice with deletion of the whole *GIPR* gene [86]. In comparison to wild-type controls, GIPR KO mice showed increased levels of the bone resorption marker urinary deoxypyridinoline and reduced levels of the bone formation markers osteocalcin and alkaline phosphatase [80].

Mouse osteoblasts, osteocytes and osteoclasts have been shown to express GLP-1R [77,87,88], and glucose-dependent expression of GLP-1R was also demonstrated in differentiating osteoblasts induced by bone morphogenetic protein-2 (BMP-2) [77]. In addition, the reaction of mouse osteoblast MC3T3-E1 cells to GLP-1 demonstrated functional effects independent of the cAMP-linked GLP-1R [89]. Briefly, GLP-1 was shown to affect the differentiation and activity of MC3T3-E1 cells via glycosylphosphatidylinositol binding receptor, inducing hydrolysis of glycosylphosphatidylinositol and increasing the activities of PI-3 kinase and MAPK in these cells [89]. They also reported that GLP-1 decreased the expression of Runt-related transcription factor 2 (Runx2) but increased that of osteocalcin without activating the canonical Wnt signaling pathway in MC3T3-E1 cells [89]. Bone marrow stem cells and adipose-derived stem cells recovered from adipose tissue were shown to express the GLP-1R [90,91,92,93], and its level of expression was reported to be upregulated during the differentiation of adipose-derived stem cells into osteoblasts in vitro [91]. GLP-1 injection was shown to have beneficial effects on bone structure, including trabecular separation and trabecular bone pattern factor (TBPf), in 10-week-old d-fructose-induced insulin-resistant and streptozotocin-induced diabetic rats [94], and 10-week-old GLP-1R KO mice showed reduced tibial and vertebral cortical bone mass and strength in comparison to wild-type controls [95]. Although GLP-1R KO mice showed no significant differences in bone mineral quantity or quality compared to wild-type controls, their collagen matrix was significantly less mature resulting in reduced bone mineral content, bone diameter, cortical thickness, and yield strength [96]. GIPR disruption was shown to induce a compensatory increase in GLP-1 secretion, and conversely suppression of GLP-1 led to a compensatory increase in GIPR expression [97,98]. A GIPR and GLP-1R double-KO mouse model was shown to have higher trabecular bone mass than wild-type controls, although cortical area, cortical thickness, and bone outer diameter were decreased [99].

## 3. Effects of Incretin on Bone Resorption

GIP has been reported to inhibit the apoptosis of both osteoblasts [80,81] and bone marrow stem cells [81]. On the other hand, GIP decreased osteoclast activity in vitro in a dose-dependent manner, and inhibited PTH and RANKL-induced bone resorption [100]. GIP infusion was also shown to inhibit bone resorption in humans [101]. These observations suggested that GIP promotes bone formation and may interfere with bone resorption. Impaired bone strength was also observed in 5-month-old GIPR KO mice, which was probably due to decreased bone formation and increased bone resorption [84], while overexpressing GIP transgenic mice showed increased expression of the bone formation marker osteocalcin and decreased expression of the bone resorption marker deoxypyridinoline, as well as a decrease in osteoclast number and an increase in bone mass [102]. In addition, daily injections of GIP for 6 weeks in ovariectomized (OVX) 8-week-old mice reduced bone loss [103]. GIPR KO mice were shown to have increased osteoclast number by histochemical analysis and a lower rate of bone formation by bone tissue morphometric analysis demonstrated, in addition to increased urinary excretion of deoxypyridinoline [80]. Conversely, histological morphometry indicated a decrease in osteoclasts and RANKL gene expression was decreased in GIPR KO mouse osteoblast cultures [85].

On the other hand, GLP-1R expression was not detected in osteoclasts [96]. High doses of GLP-1 did not affect the level of the RANKL decoy receptor osteoprotegerin (OPG) mRNA expression. In addition, GLP-1 has been reported to have no effect on osteoclast differentiation and activity [95]. The number of osteoclasts and bone resorption were reported to be higher in GLP-1R KO mice than wild-type controls [95], and μCT analysis showed that hyperlipidemic rats with subcutaneous administration of GLP-1 for 3 days had increased femoral and vertebral bone mass compared to wild-type controls [104]. These observations demonstrated that GLP-1 is associated with upregulation of OPG and osteocalcin mRNA expression, thus indicating increased bone formation. In addition, treatment of diabetic animal models with GLP-1R agonists was reported to prevent bone loss [82,105].

A high-fat diet was reported to be associated with decreased OPG/RANKL ratio and increased bone resorption resulting in reduced bone mass in Wistar rats. Treatment of these high-fat diet rats with the GLP-1R agonist, exendin-4, increased the OPG/RANKL ratio and reduced the extent of bone loss. Histological morphological analysis showed that treatment of high-fat diet rats with exendin-4 resulted in reductions in osteoclast number and eroded surface area as well as increases in osteoid area, bone mass, and trabecular bone volume compared to untreated controls maintained on high-fat diet [104]. These observations suggested that exendin-4 may prevent the harmful effects of bone defects caused by a hyperlipidemia. Treatment of 12-week-old osteoporotic OVX mice with exendin-4 for 4 weeks resulted in increased bone mass and trabecular number as determined by μCT and bone tissue morphometric analysis indicating a decrease in bone resorption. However, the administration of exendin-4 to osteoporotic OVX rats resulted in increases in femoral and vertebral BMD, and the effects of osteoclasts on bone resorption were explained by changes in calcitonin levels, which were increased by exendin-4 treatment [87]. Treatment of these 12-week-old osteoporotic OVX mice with estradiol as a positive control and with exendin-4 reduced the levels of bone resorption markers, such as serum CTX. On the other hand, the bone formation markers osteocalcin and P1NP in the rats administrated exendin-4 were higher than estradiol-treated rats [106].

Long-term treatment with high doses of exendin-4 also increased secretion of calcitonin in wild-type mice, but no such increases were observed in GLP-1 KO mice [107]. Furthermore, a single injection of exendin-4 has been exhibited increasing gene expression of calcitonin [95]. Treatment with the GLP-1R agonist, liraglutide, for 4 weeks was shown to prevent trabecular loss in 12-week-old OVX mice. Bone tissue morphometric analysis demonstrated no changes in bone formation rate, and no changes in calcitonin or sclerostin levels in these mice, indicating that liraglutide reduces bone resorption but does not affect bone formation [80]. Furthermore, the ratio of low-density lipoprotein receptor-related protein 5, which is a Wnt pathway activator, to sclerostin increased after 3 days of exendin-4 treatment [94]. GLP-1 seems to stimulate calcitonin secretion in rodents and may cause the observed changes in bone resorption, and long-term and high doses of liraglutide treatment of monkeys also did not cause C-cell hyperplasia or calcitonin secretion [107]. These observations emphasize the marked differences in the effects of GLP-1 on bone metabolism between mammalian species.

## 4. Effects of Incretin-Related Drugs on Bone Formation and Bone Resorption

### 4.1. GLP-1R Agonists

The GLP-1R agonist, exenatide, is a peptide analog of GLP-1 that has similar actions to GLP-1 but a longer plasma half-life due to its resistance to degradation by DPP-4, and is therefore characterized by both good efficacy and a long duration of action [108]. Exenatide, shares about 53% of its amino acid sequence with human GLP-1, while the much longer-acting GLP-1 analog, liraglutide, has 98% identity with human GLP-1.

GLP-1Rs have been identified in several tissues and organs, including bone, and therefore GLP-1 is thought to affect bone metabolism [109,110,111,112,113,114,115]. In preclinical studies in humans, treatment of type 2 diabetes patients with exenatide-4 for 44 weeks did not affect serum levels of bone metabolism markers and did not significantly alter BMD, even though patients showed a 6% reduction in body weight [116]. The results of two meta-analyses of randomized controlled trials by Mabilleau et al. and Su et al. examining the relations between GLP-1R agonist treatment for glycemic control and bone fracture incidence rates showed that neither exenatide nor liraglutide affected the risk of fractures in patients with type 2 diabetes compared to placebo or other antidiabetic drugs, such as glimepiride, sitagliptin, and insulin, and that exenatide increases the risk of fractures, while liraglutide reduces the risk of non-vertebral fractures, indicating that different GLP-1R agonists have different effects on fracture risk [117,118]. However, both were preliminary studies and further research will be necessary to determine whether GLP-1R agonists have favorable or unfavorable effects on risk of fractures. In addition, neither of these meta-analyses was designed specifically to assess fracture risk and included short-term randomized trials in which there were only small numbers of fractures with follow-up periods of insufficient length to allow examination of the therapeutic effects of treatment on fractures [119].

It has been reported that GLP-1R agonists increase the serum level of the bone formation marker, osteocalcin, which is expressed by osteoblasts, resulting in increases in bone mass and strength [120,121,122]. Treatment with the GLP-1R agonist, liraglutide, was shown to ameliorate ligature-induced alveolar bone resorption and reduce the number of osteoclasts on the alveolar bone surface in an animal model of periodontitis [123]. The same GLP-1R agonist, liraglutide, was shown to directly inhibit RANKL-induced osteoclast differentiation and bone resorption by inhibiting the NF-κB and MAPK signaling pathways via GLP-1R [115]. A study of the effects of GLP-1R agonism on OVX-induced osteoporosis in aged rats showed that 16 weeks of treatment with exendin-4 prevented trabecular microarchitecture deterioration and increased bone strength by inhibiting bone resorption via an increase in the OPG/RANKL ratio and promoting bone formation by increasing osteoblast-specific transcription factor expression [106]. Exendin-4 was also reported to activate osteoblasts and prevent bone loss in an OVX osteoporosis model [124]. The GLP-1R agonist, liraglutide, was reported to directly promote bone formation by the MC3T3-E1 osteoblastic cell line via activation of ERK1/2, PI3K/AKT, and cAMP/PKA/β-cat-Ser675 signaling through GLP-1Rs [125]. The GLP-1R is expressed in the osteocyte cell line, MLO-Y4, and osteocytes of the rat femur, further supporting the roles of GLP-1 in bone metabolism. Exendin-4 was shown to reduce sclerostin (SOST) mRNA expression and protein production in MLO-Y4 cells, and to reduce the sclerostin level in type 2 diabetic rats [126].

The levels of bone destruction were shown to be increased in GLP-1R-deficient mice, suggesting that osteoclast differentiation and bone resorption were inhibited by GLP-1R signaling [95]. In a mouse model of lipopolysaccharide (LPS)-induced osteoclast formation and bone resorption, we showed that co-treatment with exendin-4 significantly reduced the number of osteoclasts, the ratio of bone resorption pits, and the level of the bone resorption marker, CTX, compared to mice injected with LPS alone [65]. In addition, the exendin-4 and LPS co-treatment group showed reduced levels of RANKL and TNF-α mRNA expression compared to mice injected with LPS alone, but there were no direct effects of exendin-4 on osteoclast differentiation induced by RANKL and TNF-α, or on LPS-induced RANKL expression in stromal cells in vitro. However, co-treatment with exendin-4 and LPS resulted in the inhibition of TNF-α mRNA expression in macrophages compared to treatment with LPS alone. Taken together, these observations showed that exendin-4 reduced LPS-induced osteoclast differentiation and bone resorption by inhibiting the production of TNF-α induced by LPS in macrophages [65]. To increase the inhibitory effects, the mice in our study received daily subcutaneous injections of exendin-4 at dose of 20 μg/day for 5 days above the calvariae, corresponding to ~1 mg/kg/day, which was higher than the daily dose of 20 μg/kg/day for 4 weeks used in previous rodent studies that showed no effect [127,128]. Further studies are therefore required to examine the effects of doses corresponding to those appropriate for clinical use. As TNF-α induces osteoclast differentiation and leads to upregulation of the expression of RANKL in stromal cells, our findings outlined above implied that the inhibitory effect of exendin-4 on LPS-induced osteoclast differentiation in vivo was mediated by the inhibition of LPS-induced TNF-α expression in macrophages, thus suppressing RANKL expression induced by TNF-α in stromal cells. Tu et al. examined the effects of the GLP-1R agonist, liraglutide, in a rat model of subarachnoid hemorrhage (SAH)-induced brain injury, and reported that it attenuated the increases in levels of inflammatory mediators, including cyclooxygenase-2 (COX-2), inducible nitric oxide synthase (iNOS), TNF-α, and IL-1β [129]. These observations suggested that GLP-1R agonists may have anti-inflammatory capabilities, and may therefore inhibit inflammation-induced osteoclast differentiation. A number of studies indicated that exendin-4 can ameliorate osteoporosis in animal models of diabetes, OVX, and high-fat diet [87,94,104,105,106,130]. The underlying mechanisms responsible for these effects have been suggested to include reduction in the RANKL/OPG ratio [94,104,106], downregulation of TNF-α expression in bone [65], and a decrease in the serum calcitonin level [87,95]. Exendin-4 showed no direct effect on osteoclasts via GLP-1Rs as it showed little to no effect in vitro [65,87,106]. In addition, exendin-4 was shown to promote the osteoblastic differentiation of bone marrow stromal cells (BMSCs) via Wnt/β-catenin signaling [92]. Under inflammatory conditions in alveolar bone, exendin-4 induced osteogenic differentiation of periodontal stem cells by regulation of Wnt and NF-κB signaling and therefore downregulated bone resorption and upregulated bone formation [131]. Treatment with the GLP-1R agonist, liraglutide, was shown to directly inhibit osteoclast differentiation of both murine bone marrow-derived macrophages (BMMs) and RAW264.7 cells and bone resorption. Liraglutide inhibits NF-κB and MAPK signaling, and finally inhibits the expression of nuclear factor of activated T cells cytoplasmic 1 (NFATc1). Moreover, small interfering RNA (siRNA)-mediated GLP-1R knockdown in RAW264.7 cells reversed the inhibitory effect of liraglutide on NFATc1 expression via NF-κB and MAPK signaling [106,115]. Further studies are required to investigate the mechanisms underlying the differences in the actions of exendin-4 and liraglutide.

Orthodontic tooth movement (OTM) is induced by remodeling of alveolar bone resorption by osteoclasts and new bone formation by osteoblasts in response to mechanical loading. Root resorption also occurs along with osteoclast resorption in the process of OTM. RANKL [132,133] and TNF-α [134,135] are important factors involved in the differentiation of osteoclasts and odontoclasts during OTM, and exendin-4 reduces not only TNF-α expression [65,136] but also TNF-α-induced inflammatory reactions [137,138,139]. Increased RANKL expression in the bone of osteoporotic rodents was also shown to be ameliorated by administration of exendin-4 [94,104,106]. Therefore, we examined the effects of exendin-4 on OTM and root resorption in a mouse model and showed that high-dose exendin-4 inhibited osteoclast formation, tooth movement, and orthodontic force-induced root resorption during orthodontic loading [67]. Injection of 20 μg of exendin-4 reduced the expression of both RANKL and TNF-α mRNA in the maxillae, which was consistent with the results of histological analysis. TNF-α expression was shown to be inhibited by exendin-4 via suppression of the intracellular NF-κB pathway [140]. Root resorption is an undesirable outcome during orthodontic treatment [141]. It is important to find a way to prevent root resorption. Therefore, clinical application of exendin-4 for preventing root resorption is possible due to inhibiting odontoclast formation. As exendin-4 leads the polarization of macrophages toward the M2 phenotype, the expression level of TNF-α as an M1 phenotype-related cytokine is decreased [142]. Ma et al. reported that subcutaneous injection of OVX rats with exendin-4 for 3 months reduced the RANKL/OPG ratio by decreasing the level of RANKL expression and increasing that of OPG [106]. However, although the exendin-4 treatment group showed slight elevation of OPG mRNA expression, the effect was not statistically significant. Further long-term studies are required to elucidate the effects of exendin-4 on OPG expression in OTM, and additional monitoring is required during orthodontic treatment in diabetic patients taking exendin-4 for glycemic control. Further clinical application of exendin-4 may be possible due to its negative effect on root resorption.

### 4.2. DPP-4 Inhibitors

The elucidation of the incretin-insulin pathway prompted the development of DPP-4 inhibitors to prolong the circulating half-life of endogenous incretins to achieve glycemic control in diabetes patients, with a focus on drugs that can be taken orally. Sitagliptin was the first such DPP-4 inhibitor to become available, and received regulatory approval for clinical use in the USA in 2006 [143]. This was followed by saxagliptin and linagliptin, and alogliptin received US Food and Drug Administration (FDA) approval in 2013. The new agent, vildagliptin, is available for use in Europe but has not yet received approval in the USA.

There have been only a few human studies regarding the effects of DPP-4 inhibitors on bone. The neutral role of vildagliptin has been demonstrated in patients with type 2 diabetes who have not been treated with medication. The results showed that postprandial circulation levels of bone resorption markers and calcium homeostasis also did not change compared to baseline and placebo [144]. Several studies investigated the relations between DPP-4 inhibitors and fracture risk [145,146,147]. The results of a population-based cohort study in 3996 type 2 diabetes patients treated with DPP-4 inhibitors, including sitagliptin, vildagliptin, saxagliptin, alogliptin, linagliptin, gemigliptin, and evogliptin, as second-line antidiabetic medication in Taiwan showed that that these drugs reduced the fracture risk [147]. A meta-analysis of 28 clinical trials enrolling more than 21,000 patients with type 2 diabetes also showed that DPP-4 inhibitors reduced fracture risk compared to placebo and other antidiabetic treatments [145].

However, a retrospective cohort study using data from the Clinical Practice Research Datalink (CPRD) in the UK, previously known as the General Practice Research Database (GPRD) [https://www.cprd.com] (accessed on 25 May 2021), showed that DPP-4 inhibitor use among patients with type 2 diabetes was not associated with differences in fracture risk compared to nondiabetic controls or to patients receiving other adjusting for use of other antidiabetic drugs, such as metformin, sulfonylurea (SU), and thiazolidinedione (TZD) [146]. Several factors may have been responsible for these discrepancies between the above studies [148]. That is, data on fractures were not collected consistently in the studies included in the meta-analysis, and the follow-up period differed between studies, with a short average follow-up of 35 weeks and a long median follow-up of 5 years in the meta-analysis [146]. However, the findings were consistent with a large, randomized, placebo-controlled trial of cardiovascular outcomes associated with saxagliptin treatment reported by Scirica et al. [149]. Monami et al. reported no significant difference in fracture rate of diabetic patients receiving saxagliptin and controls receiving only placebo [145]. In their type-2 diabetes cohort study, Gamble et al. reported that patients treated with DPP-4 inhibitors showed no increase in fragility fracture risk compared to those receiving SU and insulin, and that they also showed reduced risk in comparison to those receiving thiazolidinediones [150].

As DPP-4 inhibitors prolong the effects of GLP-1 by preventing the inactivation of incretins by DPP-4, they may show similar effects on bone to GLP-1. Glorie et al. reported that the DPP-4 inhibitor, sitagliptin, ameliorated the reduction in trabecular number and increased trabecular spacing in streptozotocin-induced diabetic rats, probably via decreased bone resorption, and prevented cortical bone growth stagnation thus leading to increased femoral strength [151]. DPP-4 inhibitors are known to be effective in the treatment of type 2 diabetes by regulating insulin and glucagon secretion with few major side effects. In addition, animal studies have shown that DPP-4 inhibitors can attenuate obesity-related inflammation and atherosclerosis [152,153,154]. The results of clinical studies indicated that treatment with DPP-4 inhibitors is associated with reduced fracture risk in diabetic patients compared to other antidiabetic drugs, and the potent and highly selective DPP-4 inhibitor, sitagliptin, was also shown to improve bone density, bone quality, and bone markers [145,155,156,157].

In a mouse model of LPS-induced inflammation, we showed that osteoclast number, ratio of bone resorption area to total area, and serum level of the marker of bone resorption, CTX, were significantly reduced in mice co-treated with LPS along with the DPP-4 inhibitor, linagliptin, than in those given LPS alone. Moreover, the animals co-treated with LPS and linagliptin showed lower RANKL, TNF-α, tartrate-resistant acid phosphatase (TRAP), and cathepsin K mRNA levels than the LPS-only group. Our in vitro results showed that DPP-4 inhibitor and DPP-4 did not have direct effects on RANKL- or TNF-α-induced osteoclast differentiation or on RANKL expression induced by LPS in stromal cells. In contrast, TNF-α mRNA expression level was lower in macrophages from mice co-treated with LPS and linagliptin than from those treated with LPS alone. However, there was no difference in TNF-α mRNA expression between macrophages co-treated with LPS and linagliptin and those treated with LPS alone in vitro [64]. Kröller-Schön et al. reported that linagliptin ameliorated oxidative stress and vascular dysfunction in a rat model of LPS-induced sepsis, resulting in significant reduction in the infiltration of CD11b/c-positive cells into the vessel walls [158]. Moreover, DPP-4 inhibition by the gliptins, linagliptin, sitagliptin, and liraglutide, as well DPP-4 KO, improved survival, vascular inflammation, and vascular dysfunction in an animal model of LPS-induced endotoxemia [159]. Taken together, these observations showed that DPP-4 inhibition attenuates the effects of LPS. We reported that linagliptin inhibited LPS-induced osteoclast differentiation, bone resorption, and osteoclast-related cytokine expression. The results of several other studies showed that DPP-4 inhibitors ameliorated defects in bone metabolism by increasing bone density and bone quality, with increases and decreases in markers of bone formation and resorption, respectively [145,155,156,157]. Inhibition of DPP-4 was also shown to ameliorate inflammation-induced bone resorption, and may therefore be useful not only for glycemic control in diabetes patients but also for treatment of inflammation-induced bone resorption. We demonstrated that the DPP-4 inhibitor, linagliptin, ameliorated the increases in levels of TNF-α, RANKL, and M-CSF mRNA expression seen in mice treated with LPS. DPP-4 inhibition did not inhibit LPS-induced RANKL expression in stromal cells indicating that the observed amelioration of the LPS-induced upregulation of RANKL expression was not due to a direct effect of DPP-4 inhibitor on these cells. However, we also observed reduced RANKL expression level in calvarial bones in vivo, suggesting that other mechanisms affecting RANKL expression remain to be identified [64]. We also examined whether linagliptin could inhibit LPS-induced TNF-α expression in peritoneal macrophages, as macrophages are the major producers of this cytokine. The results indicated that linagliptin had an inhibitory effect on LPS-induced TNF-α expression by macrophages in vivo, while no decrease was observed in TNF-α mRNA expression by macrophages treated with LPS and DPP-4 in comparison to those treated with LPS alone in vitro. Although further studies are required to determine the reasons for these discrepancies between the results of in vivo and in vitro experiments, it is likely that other cell types and/or mediators are also involved in the effects of DPP-4 inhibitors. In contrast to our findings, Ervinna et al. reported that the DPP-4 inhibitor, anagliptin, prevented the induction of TNF-α expression by LPS in the human monocyte cell line, THP-1, in vitro [160]. These discrepancies between the two studies may have been due to the use of different DPP-4 inhibitors, i.e., linagliptin vs. anagliptin.

The in vivo inhibition of LPS-induced osteoclast differentiation by DPP-4 inhibitors may be mediated by the inhibition of LPS-induced TNF-α expression in macrophages with subsequent suppression of RANKL expression in stromal cells, as TNF-α has been shown to induce the expression of RANKL and M-CSF in stromal cells and to directly induce osteoclast differentiation [161]. DPP-4 inhibitors may prevent the induction of osteoclast differentiation and bone resorption by LPS by downregulation of LPS-induced TNF-α production by macrophages. The inhibition of LPS-induced osteoclast differentiation by DPP-4 inhibitors observed in vivo may be unrelated to its direct effects on the differentiation and proliferation of osteoclast precursor cells and direct effects on the expression of RANKL in stromal cells.

Using a mouse model of OTM involving the application of a nickel–titanium closed coil spring to move the first molar in the mesial direction for 12 days, we showed that local administration of linagliptin significantly reduced the amount of OTM, the numbers of osteoclasts and odontoclasts, and the extent of root resorption [66]. In addition, the levels of TNF-α and RANKL mRNA expression were also reduced in the linagliptin-treated mice in our study, consistent with the anti-inflammatory effects of DPP-4 inhibitors reported previously in a number of different types of vascular cells and immune cells [162,163]. Linagliptin has also been reported to show beneficial effects on several inflammatory diseases [64,164]. Increases in RANKL expression by osteoclasts associated with tooth movement have been reported in OPG-deficient mice, suggesting that RANKL plays an important role in osteoclast differentiation during OTM [165]. TNF receptor-deficient mice were also reported to show less tooth movement and osteoclast formation than wild-type controls, suggesting that TNF-α may enhance osteoclast-induced bone resorption during OTM [134]. Kanzaki et al. reported that local OPG gene transfer into periodontal tissue reduced osteoclast differentiation and inhibited OTM [166], and the RANKL/OPG ratio in the gingival crevicular fluid (GCF) has also been shown to play an important role in OTM [167]. Although OPG expression is not significantly affected by DPP-4 inhibition, the expression of RANKL in alveolar bone was shown to be suppressed by injection of linagliptin, but it is not yet clear whether it directly inhibits RANKL expression during OTM. Further studies are therefore necessary to determine whether linagliptin directly inhibits RANKL expression in vivo.

M1 macrophages have pro-inflammatory phenotype involving the secretion of high levels of NO and pro-inflammatory cytokines, such as TNF-α [168], while M2 macrophages have an anti-inflammation phenotype with the secretion of IL-10 and arginase-1 [127,169]. Reduction in the M1/M2 macrophage ratio was reported to inhibit alveolar bone resorption in a mouse periodontitis model [128]. In addition, an increase in the M1/M2 macrophage ratio with high level of TNF-α expression as shown to promote root resorption in rats after 7 days of OTM [170]. He et al. showed that M1 macrophage is significantly decreased after DPP-4 inhibitor treatments, the number of M2 macrophages showed no significant differences between control and DPP-4 inhibitor treated group. Therefore, the ratio of M1 to M2 macrophage was significantly decreased after treatment of DPP-4 inhibitor [171]. These observations suggest that the linagliptin-induced decrease in M1/M2 macrophage ratio may result in the inhibition of TNF-α-induced osteoclast differentiation and root resorption. Treatment with DPP-4 inhibitors was shown to result in the suppression of OTM by inhibition of osteoclast differentiation, while the decrease in root resorption was associated with inhibition of odontoclast differentiation. Moreover, DPP-4 inhibitors were also suggested to inhibit osteoclast and odontoclast differentiation by inhibiting TNF-α and/or RANKL expression. Taken together, these results indicate that greater care is required in the application of orthodontic treatments in patients with diabetes taking DPP-4 inhibitors for glycemic control.

## 5. Conclusions

**Figure 1:**
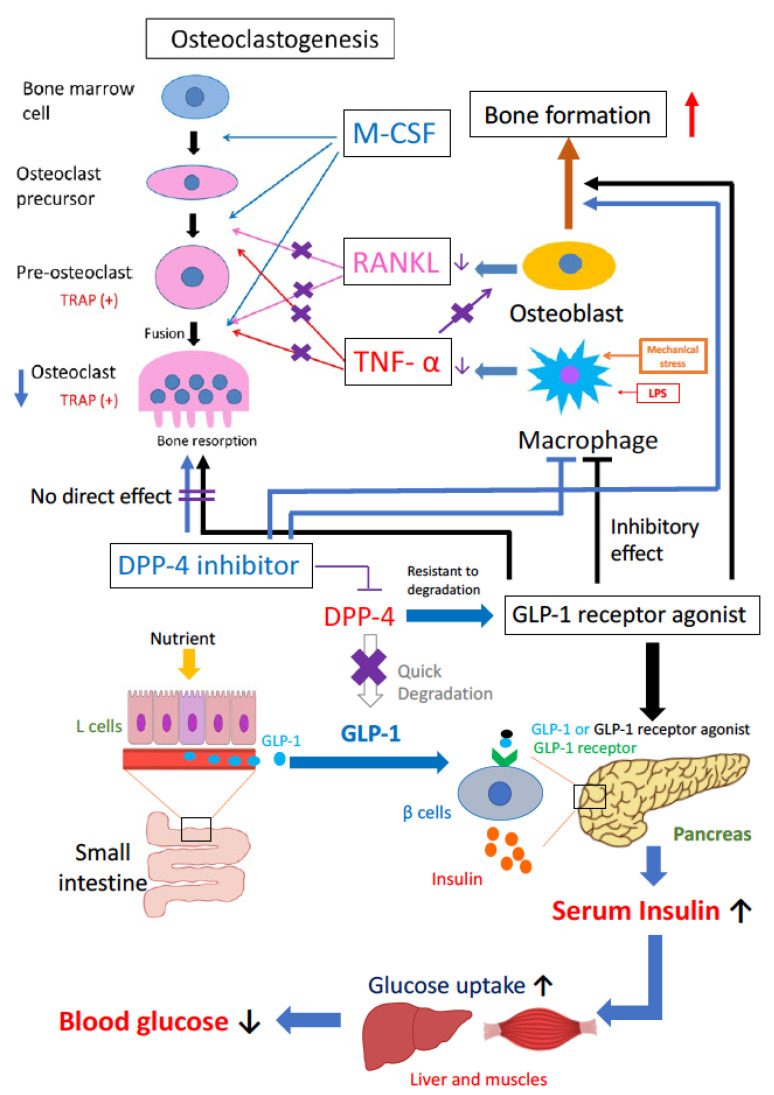
Schematic of the role of incretin-related drugs in osteoclast formation and bone resorption, and osteoblast formation and bone formation. Incretin-related drugs, GLP-1R agonists and DPP-4 inhibitors, may have positive effects on bone metabolism and increase osteoblast ability, decrease osteoclast ability and expression of inflammatory cytokines. The incretin-related drugs can inhibit inflammatory-induced osteoclast formation and bone resorption.

Incretin-related drugs, GLP-1R agonists and DPP-4 inhibitors, have positive effects on bone metabolism, increasing osteoblast function and decreasing osteoclast function. These incretin-related drugs have been shown to inhibit inflammation-induced osteoclast differentiation and bone resorption (Figure 1). However, the effects of incretin-related diabetes drugs on bone metabolism show discrepancies between in vitro and in vivo experiments, as well as between human and animal studies. Further clinical and nonclinical studies are needed to determine the effects of incretin-related diabetes drugs on bone metabolism.

## Abbreviations

BMD	bone mineral density
HbA1c	hemoglobin A1c
AGEs	advanced glycation end products
RANKL	receptor activator of nuclear factor-κB ligand
M-CSF	macrophage colony-stimulating factor
RANK	activator of nuclear factor-κB
TNF-α	tumor necrosis factor-α
PPAR-g	peroxisome proliferator-activated receptor-g
GIP	glucose-dependent insulinotropic polypeptide
GLP-1	Glucagon-like peptide-1
DPP-4	dipeptidyl peptidase-4
GLP-1R	GLP-1 receptor
ADA	American Diabetes Association
EASD	European Association for the Study of Diabetes
LPS	lipopolysaccharide
CTX	carboxy-terminal collagen crosslinks
μCT	micro-computed tomography
KO	knockout
BMP-2	bone morphogenetic protein-2
Runx2	Runt-related transcription factor 2
TBPf	trabecular bone pattern factor
OVX	ovariectomized
OPG	osteoprotegerin
SOST	sclerostin
SAH	subarachnoid hemorrhage
COX-2	cyclooxygenase-2
iNOS	inducible nitric oxide synthase
BMSCs	bone marrow stromal cells
BMMs	bone marrow-derived macrophages
NFATc1	nuclear factor of activated T cells cytoplasmic 1
siRNA	small interfering RNA
OTM	Orthodontic tooth movement
FDA	Food and Drug Administration
CPRD	Clinical Practice Research Datalink
GPRD	General Practice Research Database
SU	sulfonylurea
TZD	thiazolidinedione
TRAP	tartrate-resistant acid phosphatase
